# Supplementary material for: A network of interacting ciliary tip proteins with opposing activities imparts slow and processive microtubule growth
Source: Nat Struct Mol Biol. 2025 Jan 24;32(6):979–94. doi: 10.1038/s41594-025-01483-y (PMC12170345; doi:10.1038/s41594-025-01483-y)
Supplement: Supplementary file 1 — Reporting Summary [file 41594_2025_1483_MOESM1_ESM.pdf]

nature portfolio

Corresponding author(s): Anna Akhmanova

Last updated by author(s): Dec 31, 2024

Reporting Summary

Nature Portfolio wishes to improve the reproducibility of the work that we publish. This form provides structure for consistency and transparency in reporting. For further information on Nature Portfolio policies, see our Editorial Policies and the Editorial Policy Checklist.

Statistics

For all statistical analyses, confirm that the following items are present in the figure legend, table legend, main text, or Methods section.

n/a	Confirmed	
☐	☒	The exact sample size (*n*) for each experimental group/condition, given as a discrete number and unit of measurement
☐	☒	A statement on whether measurements were taken from distinct samples or whether the same sample was measured repeatedly
☐	☒	The statistical test(s) used AND whether they are one- or two-sided<br>*Only common tests should be described solely by name; describe more complex techniques in the Methods section.*
☒	☐	A description of all covariates tested
☐	☒	A description of any assumptions or corrections, such as tests of normality and adjustment for multiple comparisons
☐	☒	A full description of the statistical parameters including central tendency (e.g. means) or other basic estimates (e.g. regression coefficient) AND variation (e.g. standard deviation) or associated estimates of uncertainty (e.g. confidence intervals)
☐	☒	For null hypothesis testing, the test statistic (e.g. *F*, *t*, *r*) with confidence intervals, effect sizes, degrees of freedom and *P* value noted<br>*Give P values as exact values whenever suitable.*
☒	☐	For Bayesian analysis, information on the choice of priors and Markov chain Monte Carlo settings
☒	☐	For hierarchical and complex designs, identification of the appropriate level for tests and full reporting of outcomes
☒	☐	Estimates of effect sizes (e.g. Cohen's *d*, Pearson's *r*), indicating how they were calculated

Our web collection on statistics for biologists contains articles on many of the points above.

Software and code

Policy information about availability of computer code

Data collection	MetaMorph 7.10.2.240 software, Digital micrograph 3.32, Serial-EM 4.1 beta11
Data analysis	ImageJ 1.45s, ImageJ 1.53c (Fiji), ImageJ plugin KymoResliceWide v.0.4 (https://github.com/ekatrukha/KymoResliceWide), ImageJ plugin DoM_Utrecht v.1.1.6 (https://github.com/ekatrukha/DoM_Utrecht), AreTomo 1.3.3 , Cryo-CARE 0.2.2, Cryo-CARE 0.3.0, MotionCor2, EMAN2 2.91, EMAN2 2.99, IMOD 4.9.6, IMOD 4.11.20, Matlab R2022b, Matlab R2024a, Matlab scripts for g custom algorithms or softwarcurvature analysis (https://github.com/ngudimchuk/Process-PFs) that are central to the research. Python scripts for automated tomogram reconstruction and cryo-CARE denoising (https://github.com/SBC-Utrecht/cryocare-from-movies).

For manuscripts utilizing custom algorithms or software that are central to the research but not yet described in published literature, software must be made available to editors and reviewers. We strongly encourage code deposition in a community repository (e.g. GitHub). See the Nature Portfolio guidelines for submitting code & software for further information.

Data

Policy information about availability of data

All manuscripts must include a data availability statement. This statement should provide the following information, where applicable:

- Accession codes, unique identifiers, or web links for publicly available datasets
- A description of any restrictions on data availability
- For clinical datasets or third party data, please ensure that the statement adheres to our policy

Mass spectrometry data has been deposited to the ProteomeXchange Consortium via the PRIDE partner repository with the dataset identifier PXD055631. Cryo electron tomography data generated for the paper have been deposited to EMDB with the accession codes EMD-52359 and EMD-52360, and to EMPIAR with the accession codes EMPIAR-12498 and EMPIAR-12499. The EMDB and EMPIAR depositions will become publicly available once the paper is released. All data that support the conclusions are either available in the manuscript itself or available from the authors on request.

Research involving human participants, their data, or biological material

Policy information about studies with human participants or human data. See also policy information about sex, gender (identity/presentation), and sexual orientation and race, ethnicity and racism.

Reporting on sex and gender	n/a
Reporting on race, ethnicity, or other socially relevant groupings	n/a
Population characteristics	n/a
Recruitment	n/a
Ethics oversight	n/a

Note that full information on the approval of the study protocol must also be provided in the manuscript.

Field-specific reporting

Please select the one below that is the best fit for your research. If you are not sure, read the appropriate sections before making your selection.

☒ Life sciences ☐ Behavioural & social sciences ☐ Ecological, evolutionary & environmental sciences

For a reference copy of the document with all sections, see nature.com/documents/nr-reporting-summary-flat.pdf

Life sciences study design

All studies must disclose on these points even when the disclosure is negative.

Sample size	No statistical methods were used to predict the sample size. All datasets were pooled from at least three or more independent experiments. Sample size was chosen based on the reproducibility, our previous experience or the standards in the field. Data distribution was assumed to be normal but this was not formally tested. In each case, sample size, number of independent experiments and statistical tests, when used, along with p values were indicated in the figure panels, legends or within the methods section.
Data exclusions	No data were excluded from the analyses.
Replication	Each experimental condition was repeated at least three times or more unless stated otherwise. All attempts at replication were successful.
Randomization	No randomization was performed in our study as samples were not required to be allocated into experimental groups.
Blinding	Investigators were not blinded to group allocation during data collection and analyses as group allocation was not required. Data was collected immediately after each experiment was performed for each experimental condition with different proteins or different protein concentrations, and therefore blinding was not possible. The analyses were done using automated methods or using strictly defined parameters as mentioned in the methods section, therefore preventing any subjective error.

Reporting for specific materials, systems and methods

We require information from authors about some types of materials, experimental systems and methods used in many studies. Here, indicate whether each material, system or method listed is relevant to your study. If you are not sure if a list item applies to your research, read the appropriate section before selecting a response.

Materials & experimental systems

n/a	Involved in the study
☐	☒ Antibodies
☐	☒ Eukaryotic cell lines
☒	☐ Palaeontology and archaeology
☒	☐ Animals and other organisms
☒	☐ Clinical data
☒	☐ Dual use research of concern
☒	☐ Plants

Methods

n/a	Involved in the study
☒	☐ ChIP-seq
☒	☐ Flow cytometry
☒	☐ MRI-based neuroimaging

Antibodies

Antibodies used

Commerical antibodies used in the study: rabbit anti-RFP (Rockland immunochemicals, cat number 600-401-379), mouse anti-GFP (Sigma-Aldrich, cat number G1546), donkey anti-mouse IgG (H + L) IRDye® 800CW (LI-CORbioscience, cat number 926-32212) donkey anti-rabbit IgG (H + L) IRDye® 680CW (LI-CORbioscience, cat number 926-68072). Detailed information on commercial antibodies can also be found in the methods section

Validation

Commercial antibodies were validated in species mentioned above for immunofluorescence and western blots as noted on manufacturer's documentation and copied here.

Rabit anti-RFP validation according to manufactures website: This product was prepared from monospecific antiserum by immunoaffinity chromatography using Red Fluorescent Protein (Discosoma) coupled to agarose beads followed by solid phase adsorption(s) to remove any unwanted reactivities. Expect reactivity against RFP and its variants: mCherry, tdTomato, mBanana, mOrange, mPlum, mOrange and mStrawberry. Assay by immunoelectrophoresis resulted in a single precipitin arc against anti-Rabbit Serum and purified and partially purified Red Fluorescent Protein (Discosoma). No reaction was observed against Human, Mouse or Rat serum proteins.

mouse anti-GFP validation: The isotype is determined using a double diffusion immunoassay using Mouse Monoclonal Antibody Isotyping Reagents, Cat. Nos. G1546, ISO2.

secondaries validation: The antibody was isolated by affinity chromatography using antigens coupled to agarose beads. Based on ELISA, this antibody reacts with the heavy and light chains of mouse IgG, and with the light chains of mouse IgM and IgA. This antibody was tested by ELISA and/or solid-phase adsorbed to ensure minimal cross-reactivity with bovine, chicken, goat, guinea pig, horse, human, rabbit, and sheep serum proteins, but may cross-react with immunoglobulins from other species. The conjugate has been specifically tested and qualified for Western blot and In-Cell Western™ Assay applications.

Eukaryotic cell lines

Policy information about cell lines and Sex and Gender in Research

Cell line source(s)

Human embryonic kidney 239T (HEK293T) cells (Cat#CRL-3216) were obtained from ATCC

Authentication

ATCC performs short-tandem repeat profiling for cell line authentication, and no additional cell line authentication

Mycoplasma contamination

The cell line was routinely checked for mycoplasma contamination using LT07-518 Mycoalert assay and has been verified as mycoplasma free.

Commonly misidentified lines
(See ICLAC register)

The cell line used is not present in the list of commonly misidentified lines.

Plants

Seed stocks

n/a

Novel plant genotypes

n/a

Authentication

n/a
