# Supplementary material for: A network of interacting ciliary tip proteins with opposing activities imparts slow and processive microtubule growth
Source: Nat Struct Mol Biol. 2025 Jan 24;32(6):979–94. doi: 10.1038/s41594-025-01483-y (PMC12170345; doi:10.1038/s41594-025-01483-y)
Supplement: Supplementary file 3 — Uncropped gels/western blots. [file 41594_2025_1483_MOESM3_ESM.pdf]

**Saunders et al. - Source Data for ED Figure 1**

# ED Figure 1A

SII-GFP-ARMC9 FL

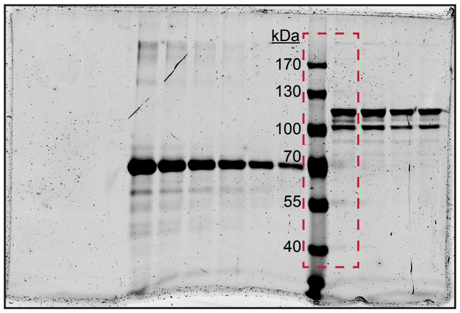


Original coomassie stained SDS-PAGE

SII-GFP-CCDC66 FL

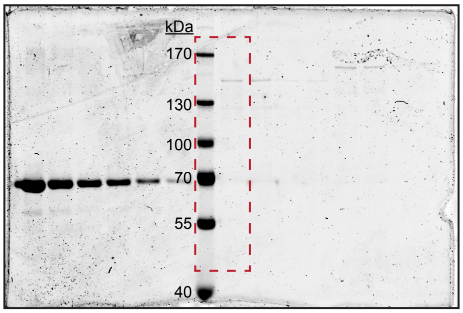


Original coomassie stained SDS-PAGE

SII-GFP-CEP104 FL

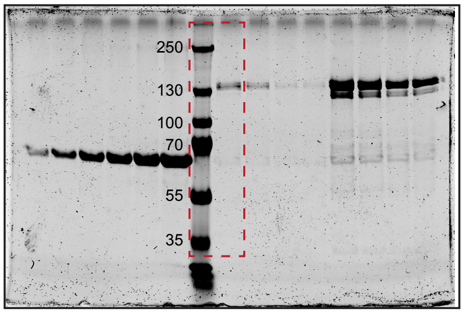


Original coomassie stained SDS-PAGE

SII-GFP-CSPP1 FL

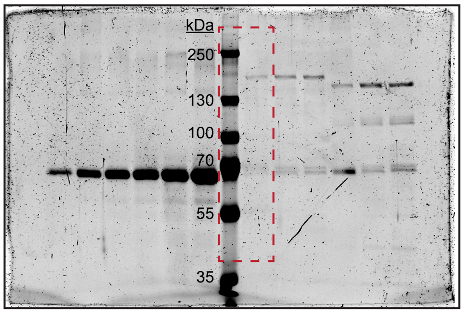


Original coomassie stained SDS-PAGE

SII-GFP-TOGARAM1 FL

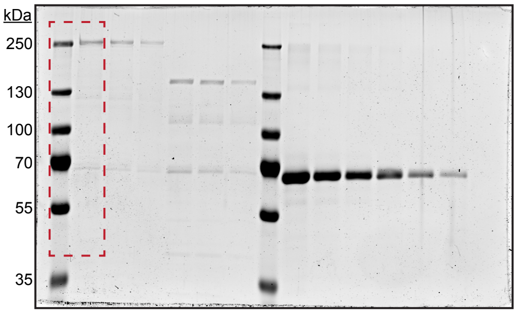


Original coomassie stained SDS-PAGE
