# Supplementary material for: A network of interacting ciliary tip proteins with opposing activities imparts slow and processive microtubule growth
Source: Nat Struct Mol Biol. 2025 Jan 24;32(6):979–94. doi: 10.1038/s41594-025-01483-y (PMC12170345; doi:10.1038/s41594-025-01483-y)
Supplement: Supplementary file 4 — Uncropped gels/western blots. [file 41594_2025_1483_MOESM4_ESM.pdf]

Saunders et al. - Source Data for ED Figure 2

ED Figure 2A left

SII-GFP-CEP104 SXNN

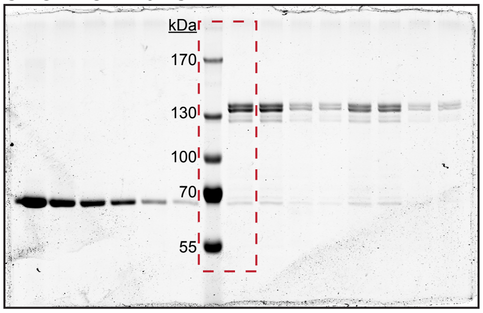

Original coomassie stained SDS-PAGE

ED Figure 2A right

SII-GFP-CEP104 ΔTOG

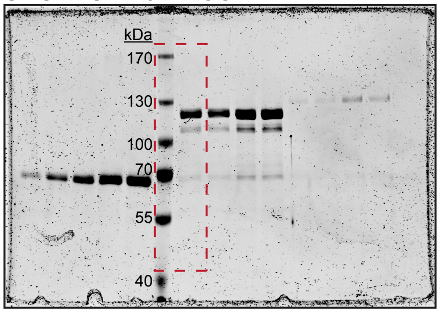

Original coomassie stained SDS-PAGE

ED Figure 2C left

Co-IP: GFP-CSPP1, input[1], IP[2]

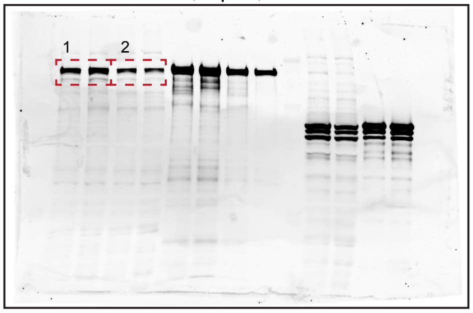

Original blot: GFP

Co-IP: mCH-CEP104 constructs, input[1], IP[2]

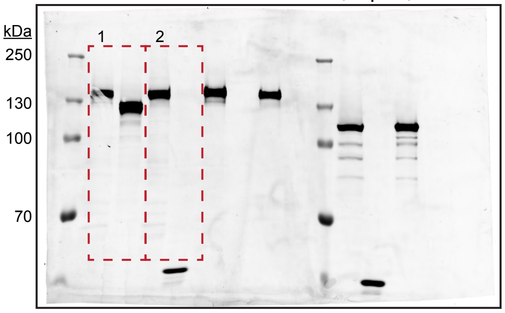

Original blot: mCherry (including Mw marker)

ED Figure 2C right

Co-IP: GFP-CCDC66, input[1], IP[2]

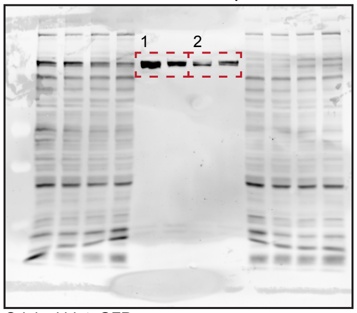

Original blot: GFP

Co-IP: mCH-CEP104 constructs, input[1], IP[2]

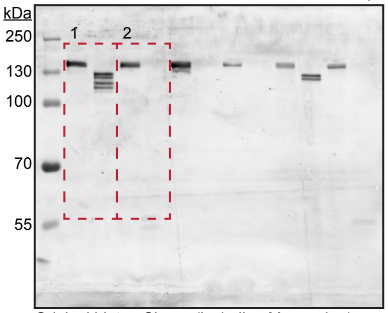

Original blot: mCherry (including Mw marker)

ED Figure 2D

SII-GFP-CEP104 ΔJR

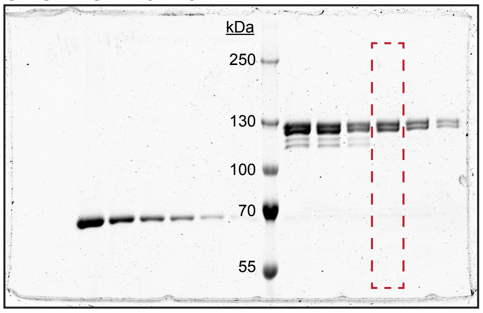

Original coomassie stained SDS-PAGE
