# Supplementary figures and images for: A network of interacting ciliary tip proteins with opposing activities imparts slow and processive microtubule growth
Source: Nat Struct Mol Biol. 2025 Jan 24;32(6):979–94. doi: 10.1038/s41594-025-01483-y (PMC12170345; doi:10.1038/s41594-025-01483-y)

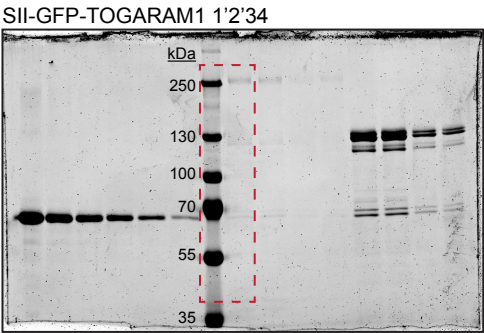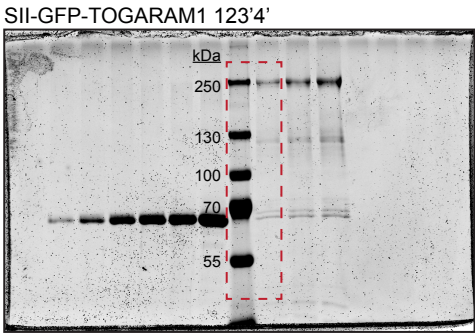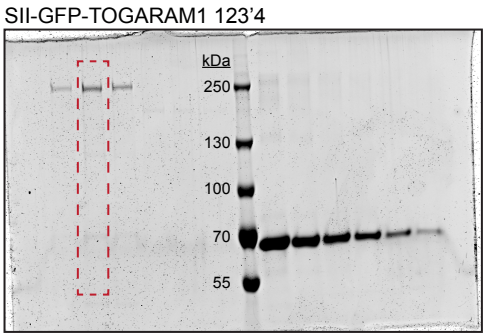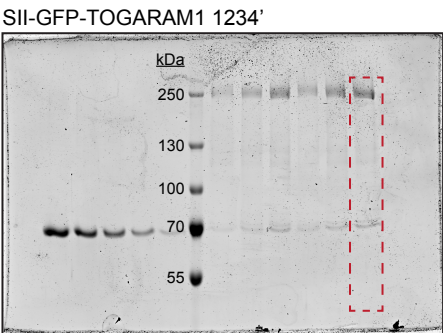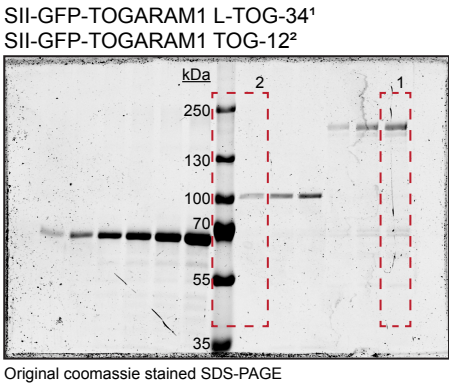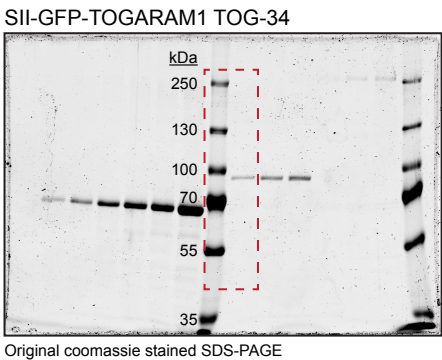

Supplement: Supplementary file 5 — Uncropped gels/western blots. [file 41594_2025_1483_MOESM5_ESM.pdf]
