# Supplementary material for: A network of interacting ciliary tip proteins with opposing activities imparts slow and processive microtubule growth
Source: Nat Struct Mol Biol. 2025 Jan 24;32(6):979–94. doi: 10.1038/s41594-025-01483-y (PMC12170345; doi:10.1038/s41594-025-01483-y)
Supplement: Supplementary file 6 — Uncropped gels/western blots. [file 41594_2025_1483_MOESM6_ESM.pdf]

Saunders et al. - Source Data for ED Figure 4

ED Figure 4C

Co-IP: GFP-ARMC9, input[1], IP[2]

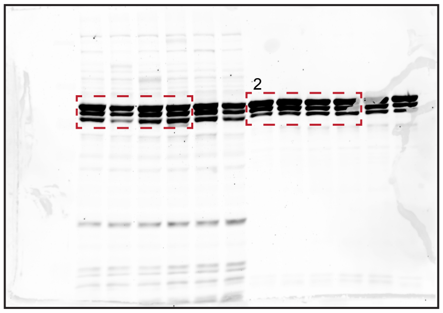

Original blot: GFP

Co-IP: mCH-CTM constructs, input[1], IP[2]

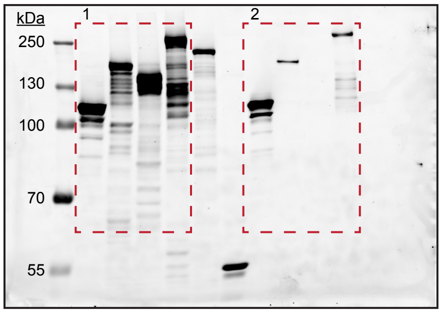

Original blot: mCherry (including Mw marker)

ED Figure 4E

Co-IP: GFP-TOGARAM1, input[1], IP[2]

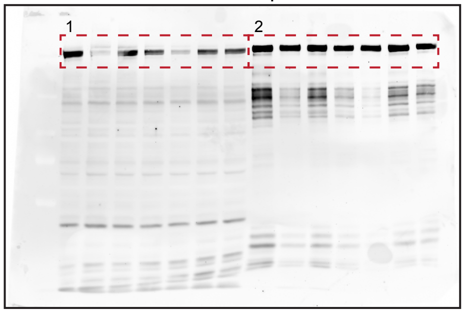

Original blot: GFP

Co-IP: mCH-ARMC9 constructs, input[1], IP[2]

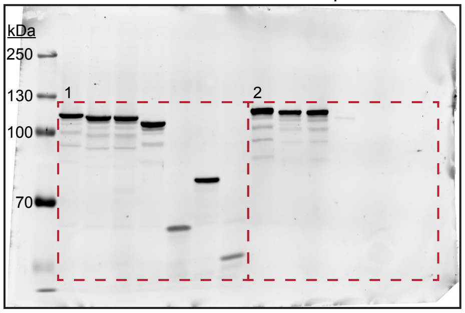

Original blot: mCherry (including Mw marker)

ED Figure 4F

Co-IP: GFP-ARMC9, input[1], IP[2]

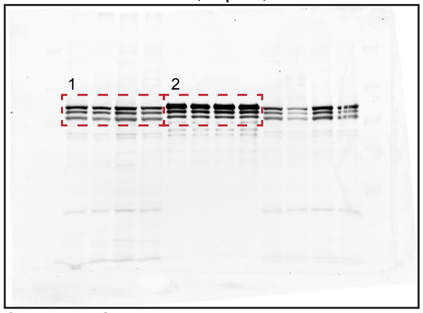

Original blot: GFP

Co-IP: mCH-TOGARAM1 mutants, input[1], IP[2]

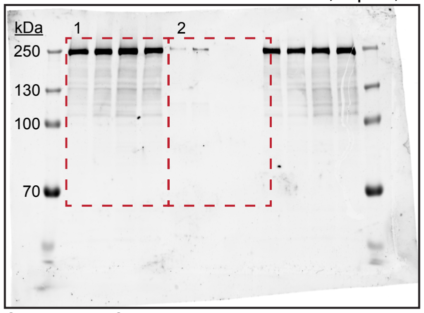

Original blot: mCherry (including Mw marker)

ED Figure 4G

Co-IP: GFP-TOGARAM1, input[1], IP[2]

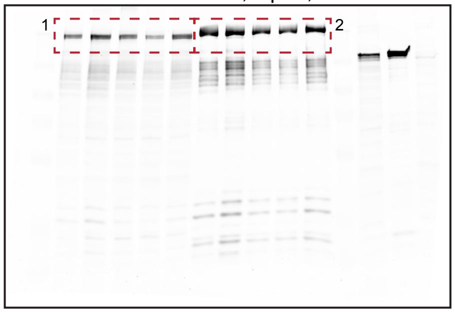

Original blot: GFP

Co-IP: mCH-ARMC9 Joubert syndrome mutants, input[1], IP[2]

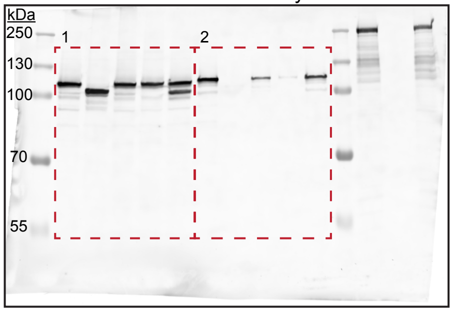

Original blot: mCherry (including Mw marker)

ED Figure 4H

Co-IP: GFP-ARMC9, input[1], IP[2]

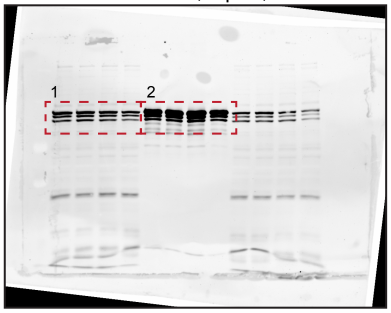

Original blot: GFP

Co-IP: mCH-TOGARAM1 Joubert syndrome mutants, input[1], IP[2]

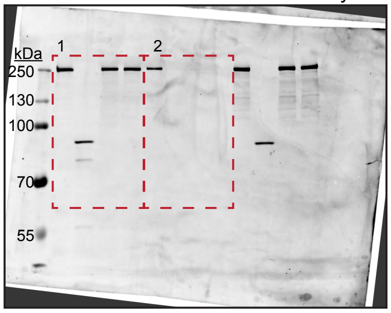

Original blot: mCherry (including Mw marker)
