# Supplementary material for: A network of interacting ciliary tip proteins with opposing activities imparts slow and processive microtubule growth
Source: Nat Struct Mol Biol. 2025 Jan 24;32(6):979–94. doi: 10.1038/s41594-025-01483-y (PMC12170345; doi:10.1038/s41594-025-01483-y)
Supplement: Supplementary file 8 — Uncropped gels/western blots. [file 41594_2025_1483_MOESM8_ESM.pdf]

Saunders et al. - Source Data for ED Figure 6

ED Figure 6A

Co-IP: GFP-CEP104, input[1], IP[2]

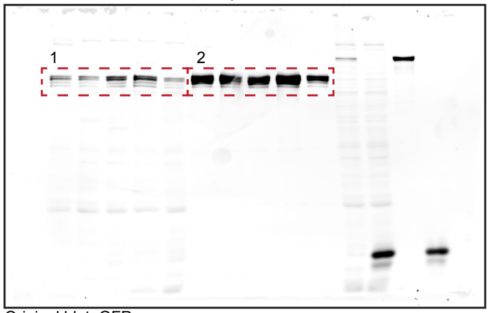

Original blot: GFP

Co-IP: mCH-TOGARAM1 consrtucts, input[1], IP[2]

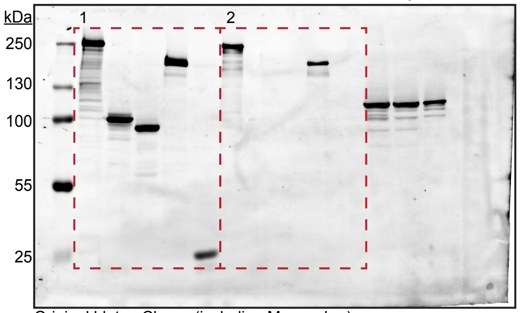

Original blot: mCherry (including Mw marker)

ED Figure 6B

Co-IP: GFP-CEP104, input[1], IP[2]

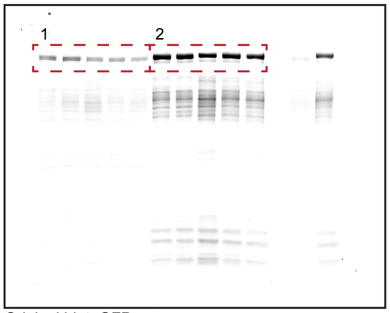

Original blot: GFP

Co-IP: mCH-TOGARAM1 consrtucts, input[1], IP[2]

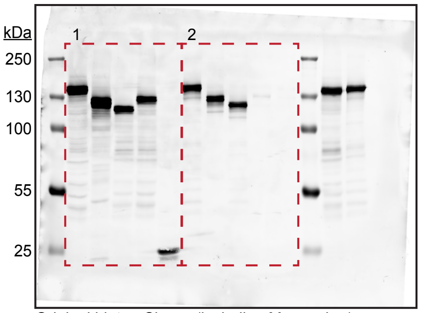

Original blot: mCherry (including Mw marker)
